# Examining influential factors in providers’ chronic pain treatment decisions: a comparison of physicians and medical students

**DOI:** 10.1186/s12909-015-0441-z

**Published:** 2015-10-01

**Authors:** Nicole A. Hollingshead, Samantha Meints, Stephanie K. Middleton, Charnelle A. Free, Adam T. Hirsh

**Affiliations:** Department of Psychology, Indiana University – Purdue University Indianapolis, 402 N Blackford, Indianapolis, IN 46202 USA

**Keywords:** Decision-making, Pain management, Virtual human, Chronic pain

## Abstract

**Background:**

Chronic pain treatment guidelines are unclear and conflicting, which contributes to inconsistent pain care. In order to improve pain care, it is important to understand the various factors that providers rely on to make treatment decisions. The purpose of this study was to examine factors that reportedly influence providers’ chronic pain treatment decisions. A secondary aim was to examine differences across participant training level.

**Methods:**

Eighty-five participants (35 medical students, 50 physicians) made treatment decisions for 16 computer-simulated patients with chronic pain. Participants then selected from provided lists the information they used and the information they would have used (had it been available) to make their chronic pain treatment decisions for the patient vignettes.

**Results:**

Frequency analyses indicated that most participants reported using patients’ pain histories (97.6 %) and pain description (95.3 %) when making treatment decisions, and they would have used information about patients’ previous treatments (97.6 %) and average and current pain ratings (96.5 %) had this information been available. Compared to physicians, medical students endorsed more frequently that they would have used patients’ employment and/or disability status (*p* < 0.05). A greater proportion of medical students wanted information on patients’ use of illicit drugs and alcohol to make treatment decisions; while a greater proportion of physicians reported using personal experience to inform their decisions.

**Discussion:**

This study found providers use patients’ information and their own experiences and intuition to make chronic pain treatment decisions. Also, participants of different training levels report using different patient and personal factors to guide their treatment decisions.

**Conclusions:**

These results highlight the complexity of chronic pain care and suggest a need for more chronic pain education aimed at medical students and practicing providers.

## Background

Chronic pain is a significant public health concern globally and in the United States, where it affects over 100 million Americans [1–3]. Chronic pain is the leading cause of healthcare utilization, and treatment can include a variety of prescription medications (e.g., opioids, nonsteroidal anti-inflammatories [NSAIDS]), over-the-counter medications (e.g., acetaminophen), and non-pharmacological interventions (e.g., physical therapy, diet and exercise) [4–6]. Despite high levels of healthcare utilization, many patients report inadequate pain management [7, 8]. Opioid medications have received particular attention in discussions regarding optimal chronic pain care, given their abuse potential and mixed support for their long-term effectiveness [9, 10].

Inadequate chronic pain management may be related to the inherent challenges of treating chronic pain [11]. Optimal chronic pain management is constrained by the fact that treatment guidelines are often inconsistent across specialty areas, give insufficient consideration to common comorbidities (e.g., depression), and lack strong supporting evidence [12–14]. Further complicating chronic pain management are providers’ concerns about dependence and abuse of opioid medications [15]. There is also a lack of training for chronic pain management in medical school and residency programs, which may be partly attributable to the mixed evidence base noted above, as well as the challenges of curriculum reform and a lack of educational resources [16, 17]. Consequently, it is unsurprising that providers report low satisfaction and lack confidence in treating chronic pain [18].

Given this ambiguous and challenging context, it is important to understand how providers make chronic pain treatment decisions. Medical decision-making is a complex process that involves synthesizing multiple sources of information [19]. Chronic pain treatment decisions rely on the synthesis of subjective information (e.g., pain ratings), objective findings (e.g., imaging results), social concerns (e.g., medication misuse), and comorbid conditions (e.g., depression); “non-medical” factors (e.g., patient sex and race, provider characteristics) have also been shown to influence the decision-making process [20–23]. Little is known about the specific factors providers report using to inform their chronic pain treatment decisions. Better understanding of these factors will contribute to current efforts to enhance evidence-based chronic pain treatment and will inform future educational efforts to improve chronic pain management. To our knowledge, no published empirical studies have examined the factors that healthcare providers report using to inform their chronic pain treatment decisions.

In addition to better understanding pain decision-making, as a whole, it is unclear whether groups of providers use similar or different factors to guide their decisions. Training level may influence providers’ treatment decisions. For example, practicing physicians often rely on clinical experience to guide decisions, which is a source of information not yet available to medical trainees who are nevertheless involved in patient care [24].

In this planned secondary data analysis of a recently completed study, we sought to explore the factors that providers reportedly use to guide their chronic pain treatment decisions, as well as the factors they would have used, had that information been available, to inform their decisions. We did not have any directional a priori hypotheses for this portion of the study. We also examined differences between medical students and physicians in their endorsement of factors that influence their decisions. For these analyses, we hypothesized that, compared to medical students, physicians would be more likely to endorse using their personal experience to inform their chronic pain treatment decisions.

## Methods

### Participants

We sought to recruit approximately 100 practicing providers (physicians, residents, nurse practitioners) and medical trainees (medical students, graduate nursing students) for the purposes of the primary study, of which the current paper is a secondary data analysis. We determined this sample size based on our previous work using a similar design [25–27]. Participants were recruited from a large Midwestern metropolitan academic medical center via posted flyers, email, and word of mouth. We recruited participants from September 2011 through January 2012.

### Study design and procedures

Detailed description of the virtual human patients and online study methods can be found in a previous paper (see [21]). Participants made pain treatment ratings for 16 virtual human patients presenting with low back pain from an injury sustained one year ago after lifting a heavy object. Patients were described as having pain in their lower back, which limits their ability to perform normal daily activities. Patients were noted to be open to any pain treatment options, to have no contraindications for treatment, and to lack other significant health problems. In order to enhance task realism, text vignettes also contained information about patients’ physiological status (e.g., blood pressure, respiration), which varied across patients but was always within normal limits. With the exception of physiological data, all of the above clinical information was held constant across the patients.

The patients’ sex, race, and mental health status were systematically balanced across the 16 vignettes. The vignettes included information on the patients’ sex (male, female) and race (White, Black) by showing a representative facial image that displayed a standardized and empirically-validated facial expression of pain [28]. The text vignettes also contained information about the patients’ mental health status (depressive symptoms present or not present over the past 6 months).

For each patient, participants indicated their likelihood of using pharmacological (e.g., opioid medication) and non-pharmacological (e.g., physical therapy) treatment options; these ratings were made on separate 0 (not at all likely) to 100 (extremely likely) visual analogue scales. After making chronic pain treatment decisions for all 16 vignettes, participants selected from a provided list the factors they used to make their treatment decisions and the factors they would have used to guide their treatment decisions had the information been provided (see Measures below). The study took approximately one hour to complete, and participants were compensated with a gift card. Study procedures were approved by Indiana University – Purdue University Indianapolis’s review board (IRB #1102004842) in accordance with the provisions of the Declaration of Helsinki.

### Measures

#### Demographic questionnaire

Participants provided information about their sex, age, and race/ethnicity. They also indicated their training level (e.g., physician or medical student). Medical students indicated their training year, and physicians indicated their years of professional experience and specialty area.

#### Information used questionnaire

Participants indicated which of the following factors informed their chronic pain treatment decisions for the 16 patient vignettes: (1) Pain history (e.g., duration of pain, cause of pain, prior treatment), (2) Patients’ description of the pain (e.g., location, level of interference with activities), (3) Patients’ facial expressions, (4) Patients’ demographic characteristics (e.g., sex, race, age), (5) Patients’ vital sign values, (6) Patients’ mental health symptoms, (7) Your own personal experience in managing pain and/or interacting with patients with pain, and (8) Your intuition. These factors were included in the questionnaire based on prior research [26, 27, 29], clinical guidelines for chronic pain management, [5] and investigators’ clinical experience, which included a clinical health psychologist, a health communications researcher, and two practicing physicians. Participants could select as many factors as applicable.

#### Information would have used questionnaire

Participants indicated the factors they would have used to inform their chronic pain treatment decisions, had the information been provided. The following factors were listed: (1) Patients’ previous experience with the treatment options, (2) Patients’ rating of their average pain over the past week, (3) Patients’ rating of their current pain, (4) Patients’ social support, (5) Patients’ use of alcohol, (6) Patients’ use of illicit drugs, (7) Patients’ vocal expressions (e.g., tone of voice, rate of speech), (8) Patients’ description of pain complaint in his/her own words, (9) Patients’ history of treatment adherence, and (10) Patients’ employment and/or disability status. These items were selected as reflecting the different types of information providers may request from patients during a clinical encounter. Participants could select as many factors as applicable.

### Statistical analyses

Frequency analyses were conducted to characterize participants’ responses to the “Information used” and “Information would have used” questionnaires. Participants were coded as a medical student or physician based on the information they provided on the demographics questionnaire. Fisher’s exact tests were used to examine training level differences (student vs. physician) in participants’ responses to the questionnaires.

## Results

### Participants’ characteristics

For these analyses, we included the 85 participants who reported being a medical student or physician. Of these participants, 56 % were male and 44 % were female. The majority of participants were non-Hispanic (97 %). Approximately 66 % were White, 24 % were Asian, 4 % were Black, 1 % was Native American, and 5 % did not report their race/ethnicity.

The sample included 35 medical students and 50 physicians. The average age of the medical students was 25 years (SD = 2.7 years). Medical students reported being in the following stage of medical education: first year (*n* = 1), second year (*n* = 17), third year (*n* = 12), and fourth year (*n* = 4); 2 participants did not report their training year. The average age of the physicians was 29 years (SD = 5.1 years). Physicians reported an average of 3 years (SD = 9.7 years) of professional experience and reported working in the following specialty areas: family/internal/primary care medicine (*n* = 23), combined internal medicine and pediatrics (*n* = 9), surgery (*n* = 3), pediatrics (*n* = 2), urology (*n* = 2), and infectious disease (*n* = 1); 10 participants did not report their specialty area. The sex, ethnic, and racial composition did not significantly differ between medical students and physicians (all *p* values > 0.05).

### Information used to inform chronic pain treatment decisions

The majority of participants reported using the patients’ pain history (97.6 %) and patients’ pain description (95.3 %) to inform their chronic pain treatment decisions (Table 1). Approximately 90 % of the sample reported using patients’ vital signs and mental health status. Many participants reported using their own personal experience in managing and/or interacting with patients with pain (89.4 %) and their intuition (88.2 %) to guide chronic pain treatment decisions for the vignettes. Approximately 85 % reported using the patients’ facial expression. Over half of the participants (62.4 %) also indicated they used patients’ demographic characteristics (i.e., sex, race, age) to inform their decisions.

**Table 1:** Percent of participants who endorsed using the following factors to guide chronic pain treatment decisions

Information used	Percent	Information would have used	Percent
Patients’ pain history	97.6 %	Previous treatment experiences	97.6 %
Patients’ pain description	95.3 %	Average pain rating over the past week	96.5 %
Mental health symptoms	91.8 %	Current pain rating	96.5 %
Patients’ vital signs	90.6 %	Use of illicit drugs	95.3 %
Your own personal experience	89.4 %	Pain description in own words	94.1 %
Your intuition	88.2 %	History of treatment adherence	92.9 %
Patients’ facial expression	84.7 %	Use of alcohol	90.6 %
Patients’ demographics	62.4 %	Employment/Disability status	84.7 %
		Vocal expression	82.4 %
		Social support	71.8 %

Participants could select more than one factor; thus, the percentages do not sum to 100

### Information would have used to inform chronic pain treatment decisions

Participants frequently endorsed wanting information on the patients’ use of illicit drugs (95.3 %) and alcohol (90.6 %; Table 1). In addition, the majority of participants reported they would have used information on the patients’ previous experiences with treatment (97.6 %) and ratings of current (96.5 %) and average pain over the past week (96.5 %) had that information been available. Participants also reported they would have used the patients’ personal (in his/her own words) descriptions of their pain complaint (94.1 %), patients’ history of treatment adherence (92.9 %), and patients’ vocal expressions (82.4 %). Relatively fewer, but still a majority of, participants also wanted information about social factors, such as patients’ employment and/or disability status and social support (84.7 and 71.8 %, respectively).

### Participant group differences

**Fig. 1:**
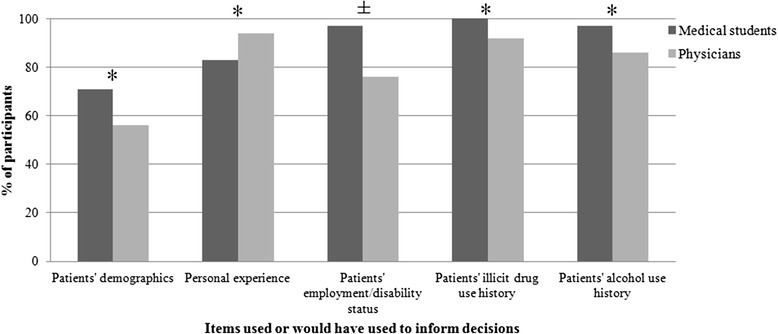
Training-level differences. *p < .05; ± refers to differences that were notable in magnitude but did not reach statistical significance. Each bar represents the percent of participants within each training level who endorsed using each factor in their chronic pain treatment decisions

Training level differences (medical student vs. physician) were examined in participants’ responses to the “Information used” and “Information would have used” questionnaires (notable differences displayed in Fig. 1).

Fisher’s exact test analyses of the “Information Used” data found physicians and medical students endorsed using similar factors when making their treatment decisions (all p values >0.10). While the differences were non-significant, there was a notably larger proportion of medical students (71 %) than physicians (56 %) who endorsed using patients’ demographics to inform their decisions, whereas a larger proportion of physicians (94 %) than medical students (83 %) reported using personal experience in their decision-making.

Compared to physicians, significantly more medical students indicated that they would have used information on patients’ employment and/or disability status (*p* = .012; students = 97 %, physicians = 76 %). There was also a non-significant yet greater proportion of medical students who indicated they would have used patients’ illicit drug (students = 100 %, physicians = 92 %) and alcohol (students = 97 %, physicians = 86 %) use history had that information been available.

## Discussion

Healthcare providers report that chronic pain is difficult to manage, in part, due to numerous and conflicting treatment guidelines that lack evidence-based support [12–14], inadequate training [16], and concerns about opioid medications [15]. Given the complexity of chronic pain management, it is important to better understand how providers make treatment decisions for patients with chronic pain. We sought to understand the factors that medical students and physicians reported using and would have used when making chronic pain treatment decisions, as well as differences between medical students and physicians. We found that participants endorsed using patients’ pain information and histories as well as their own intuition and experience to inform their treatment decisions for computer-simulated patients. Additionally, medical students, compared to physicians, reported using patients’ demographics and endorsed wanting more information on patients’ employment/disability status and substance use history; whereas a greater proportion of physicians than medical students reportedly used personal experience to guide their decisions.

The majority of participants reported using patients’ pain history and pain description to help guide their chronic pain treatment decisions. This was an expected finding given that such information is clinically instructive and commonly used to inform pain assessment and treatment decisions [4]. The majority also endorsed using patients’ mental health status when making their treatment decisions. There is a high comorbidity rate between chronic pain and depression [30], and depression is associated with poorer treatment outcomes and increased risk of opioid misuse among patients with chronic pain [31, 32]. Therefore, it is reasonable for clinicians to consider a patient’s mental health status when making chronic pain treatment decisions. Unfortunately, when faced with the question of how such clinical information should be incorporated into the decision-making process, clinicians have few resources on which to rely. There is a lack of evidence-based guidelines for dual treatment of chronic pain and depression [33, 34]. This is an area in clear need of further study, in order to provide better guidance for providers and medical trainees’ management of chronic pain.

Also noteworthy is the fact that more than half of participants reported using patients’ demographic characteristics in their chronic pain treatment decisions. While patients’ age and sex can sometimes be relevant to chronic pain care – for example, to predict risk of medication misuse and/or adverse effects [35, 36] – there is limited evidence beyond these items to support using patients’ sex, race, or age when making chronic pain treatment decisions [37–40]. On the other hand, our results are consistent with a large body of literature indicating that pain treatment varies across patient demographic groups [25, 27, 41, 42], suggesting that providers often incorporate a patient’s sex, race, and age into their treatment decision-making process. This may be particularly true for medical students who more frequently endorsed using patients’ demographic characteristics than did physicians. More broadly, this finding provides further evidence that providers often rely on non-medical factors and/or make unsystematic treatment decisions across patient populations [43, 44]. This finding highlights the need to educate both medical students (during medical school) and physicians (via continuing education) about the presence of pain disparities and discuss inappropriate uses of patients’ demographics when making clinical decisions for chronic pain.

We also queried participants about the information they would have used to inform their chronic pain treatment decisions had that information been available. The majority reported that they would have used information on patients’ previous experiences with treatments, average pain rating over the past week, and current pain rating had this information been available. Per clinical guidelines for chronic low back pain, providers should ask about patients’ previous treatments in order to assess their use of and clinical response to first-line medications (e.g., acetaminophen, NSAIDS) before considering more aggressive treatments (e.g., opioids) [5]. While participants reported they would have used patients’ average and current pain rating, other investigations found that this information plays a minor role in treatment decisions. For example, a retrospective study of acute pain in the emergency department found that patients’ pain intensity ratings played an insignificant role in providers’ decision to administer opioid medications [45]. The fact that pain ratings played such a minor role in an acute pain context, when they should probably have the most pronounced impact on providers’ treatment decisions, suggests that pain ratings exert even less of an influence on chronic pain treatment decisions. Furthermore, a number of investigations found that patients’ pain complaints are often discounted when objective diagnostic evidence is lacking and/or psychosocial factors are present (e.g., depression), as is commonly the case with chronic pain [46]. Future research should explore this discrepancy between providers’ expressed versus demonstrated use of patient pain ratings when making treatment decisions.

Given providers’ concerns about opioid abuse and dependence, it is not surprising that the majority of participants reported wanting more information about patients’ illicit drug and alcohol use history. Bhamb, et al. [47], found primary care providers were uncomfortable prescribing opioids to patients with past or current substance use problems. Although substance use is a risk factor for misuse of opioid medications in pain patients [35, 48–50], it is not an absolute contraindication for initiation of opioid therapy or other common pain treatments [51]. Over-emphasis on substance use could negatively affect clinical care and patient outcomes, as it may lead providers to wrongfully “profile” certain patients who have a history of substance abuse but who also have numerous protective factors. For instance, Dunbar and Katz [52] tracked twenty patients with a history of substance abuse and found that patients with a stable family or support system were less likely to abuse opioid medications. Thus, information on patients’ social support is likely to be important when considering patients’ substance use history and potential to abuse opioid medications.

Independent of concerns about opioid misuse, a large body of literature indicates that social support is an important factor in the functional status of patients with chronic pain [53]. More generally, social support buffers against the deleterious psychosocial effects of chronic pain [54]. Despite its importance, social support was among the lowest endorsed factors on the “Information would have used” questionnaire, suggesting a relative lack of understanding of its importance in the context of chronic pain management. Medical and continuing education should highlight the role of social support in chronic pain outcomes and emphasize its importance in chronic pain treatment decisions. Additionally, integrating person-centered care approaches that elicit information about patients’ social support system or lack thereof can strengthen the patient-provider relationship and lead to better chronic pain outcomes [55].

Medical students and physicians reported using similar factors to make their chronic pain treatment decisions, but there were a few noteworthy differences. Compared to physicians, medical students wanted significantly more information on patients’ employment and/or disability status. A greater proportion of medical students also wanted information on patients’ illicit drug and alcohol use. These results may reflect medical students’ desire for as much information as possible when making treatment decisions. Novice decision-makers tend to want more information and to use a deliberate decision-making approach, whereas experts are more likely to “size up” a situation quickly and to use their previous experiences to guide decision-making [24]. Indeed, this interpretation is consistent with our hypothesized finding that a greater proportion of physicians than medical students reported using personal experience to guide their decisions. It is also possible that information about patients’ employment/disability and substance use is more salient to medical students, as they are more likely to interact with socioeconomically disadvantaged patients during rotations than are established physicians working in a medical facility [56]. Finally, medical students may also rely on patients’ social and substance use history as indicators of risk for medication misuse, particularly opioids. Opioid misuse is a significant concern expressed by medical students when treating chronic pain [57] and education about opioids, including opioid addiction, is often not provided in medical schools [58].

A notable strength of this investigation is our use of standardized patient images and clinical text vignettes to maximize experimental control and enhance realism. However, several limitations should be discussed. Participants self-reported the factors that influenced their chronic pain treatment decisions. Accurate self-report of these factors requires decision-making awareness, which likely varied across individual participants [21, 29, 59]. The “Information used” and “Information would have used” questionnaires listed an array of relevant biopsychosocial factors, but it was not an exhaustive list of all possible factors that could influence chronic pain treatment decisions. As such, factors that participants deemed pertinent to the decision-making task could have been overlooked. Future investigations could incorporate other methods, such as qualitative interviews, to further elicit factors used in decision-making and to uncover how this information guides decisions. Finally, the generalizability of the findings may be limited due to the few provider types and single geographic area sampled in this investigation.

## Conclusions

This was one of the first investigations to examine the factors that medical students and physicians report using to make chronic pain treatment decisions. We found that providers prioritize patient information as well as personal experience and intuition when making chronic pain treatment decisions. Medical students were particularly interested in acquiring additional information on patients’ employment/disability status and to a certain extent substance use history, whereas a greater proportion of physicians reported using personal experiences to inform their decisions. This study highlights the complexity of chronic pain management and suggests a need for medical and continuing education efforts to more strongly focus on systematic, evidence-based clinical decision-making in this context.
